# The postnatal expression of transcripts and proteins in the corpus callosum, as well as its myelinization, is affected by the congenital absence of AQP4

**DOI:** 10.1007/s13105-026-01173-3

**Published:** 2026-03-31

**Authors:** Francisco Mayo, Lourdes González-Vinceiro, Laura Hiraldo-González, Ismael Torres-Rubio, Claudia Calle-Castillejo, Elaheh Sobh-Doush, Reposo Ramírez-Lorca, Miriam Echevarría

**Affiliations:** 1 Instituto de Biomedicina de Sevilla, IBiS/Hospital Universitario Virgen del Rocío, CSIC/Universidad de Sevilla, Sevilla, 41013 España; 2 Departamento de Fisiología Médica y Biofisíca, Facultad de Medicina, Universidad de Sevilla, Sevilla, 41009 Spain

**Keywords:** Aquaporin-4, Endfeet astrocytes, Microglia, Oligodendrocyte, Corpus callosum, Myelination

## Abstract

During postnatal development in mice there is a marked switch in the expression of AQP4 from white to grey matter regions. A microglial population, CD11c+, which has been shown to be involved in normal postnatal development of the corpus callosum (CC), prolongs its expression in this tissue in the absence of AQP4. Here, we investigated the correlation between the levels of AQP4 expression during the early postnatal period and the expression of marker genes related to oligodendrogenesis in the mouse CC. A microarray transcriptomic analysis of the CC of wild-type (WT) and AQP4-KO (KO) mice was performed, validation of differentially expressed genes was done by RT-qPCR, and protein expression was analyzed by immunofluorescence. Overexpression of genes associated with microglia and astrocytes and inhibition of genes associated with mature oligodendrocytes were observed in the KO animal compared to the WT. GFAP and CD11c signals were significantly higher in the CC of the KO animal, as was the number of OPCs (OLIG2+/PDGFRa+). However, the number of mature oligodendrocytes (OLIG2+/CC1+) was reduced in the KO mice, indicating a failure of the oligodendrogenesis process that results in a significant reduction in the number of myelinated axons in the CC of the KO animal. This mouse model of congenital AQP4 deficiency, which shows defects in the maturation of its oligodendrocytes in the CC, provides insight into the role of AQP4 in demyelinating pathology and could help in the development of new diagnostic and/or therapeutic strategies for such diseases.

**Supplementary Information:**

The online version contains supplementary material available at 10.1007/s13105-026-01173-3.

## Keypoints

- Astrocytes and CD11c+ microglia are overexpressed in the CC of AQP4-KO mice.
- Conversion of OPCs to mature oligodendrocytes is restricted in brains lacking AQP4.
- Oligodendrogenesis fails in the CC when AQP4 is absent.
- A low number of myelinated axons has been found in the CC of AQP4-KO mice.

**Supplementary Information:**

The online version contains supplementary material available at 10.1007/s13105-026-01173-3.

## Introduction

The corpus callosum (CC) is the largest tract of axonal fibers in the mammalian brain and one of the main routes of interhemispheric communication [1, 2]. Its mission is to facilitate complex functions associated with bilateral sensory integration and cognitive activity, such as speech development or problem solving [3]. The development of the CC begins in the embryonic period when, after fusion of the hemispheres, structures in the midline generate a route for the interhemispheric passage of axons [4, 5]. In mice, at stage E15, a series of specific glial cells contribute to the growth of the axons that cross the midline of the CC and dictate the organizational model of these axonal fibers [6]. A significant amount of initial projections that form the CC will be refined [1], while others will be preserved intact. In this process, the participation of diverse set of central nervous system (CNS) cells (oligodendrocytes, astrocytes and microglial) will be necessary, which will fundamentally modify both the morphology and functionality of the fiber bundles that make up the CC and which will end with a phase known as myelination [3, 7–10]. Myelination refers to the formation of myelin sheaths, that surround the axons of neurons providing them with a more efficient function of electric conduction in the brain. This process begins during the late fetal period, beginning with the proliferation of neural stem cell derived oligodendrocyte precursor cells (OPCs), which differentiate into immature oligodendrocytes (pre-OLs) and finally differentiate into mature oligodendrocytes (OLs) that contact neuronal axons and start the production of myelin sheath [8–13]. Expression of certain factors, while inhibition or silencing of some elements, would initiate and influence on the transcriptional program that promotes proliferation of OPCs and differentiation into mature cells fully capacitated to myelinate neuron axons. However, despite that oligodendrocytes are the main cells responsible for myelin production, their function is strongly influenced and determined by other glial cells, namely astrocytes and microglia [11, 12].

Fibrous astrocytes, abundant in the CC at early ages, align along the myelinated fibers [13], connect with oligodendrocytes, and help to transport to the latter, lipids, and other elements necessary in the synthesis of the myelin [11, 14]. Factors released by astrocytes, such as PDGFα, BDNF and IGF1 [15–17], are key in promoting the survival, migration and differentiation of OPCs. Interestingly, recent evidences had shown that OPCs utilize vessels for distribution throughout the CNS, involving direct contact with the abluminal endothelial surface [18]. But regulation of appropriate and timely vascular detachment of OPC is equally important for permitting OPC differentiation and proper CNS myelination. In that respect adequate placement of perivascular astrocyte endfeet while forming the blood brain barrier (BBB), and release of astrocyte-derived semaphorins (sema)-3a and sema-6a, become crucial for repealing OPCs from the brain vessels, a step necessary for subsequent differentiation of OPC into mature OLs [19]. Also after birth, a CD11c+ microglial cell population has recently been shown to play a critical role in supporting oligodendrogenesis, which is necessary for postnatal CNS development and myelination, and that is achieved by releasing factors such as IGF1, GM-CSF and Osteopontin (*Spp1*) [20–22].

In the brain, AQP4 is expressed in ependymal cells and in the foot processes of perivascular astrocytes that help form the BBB. It is also expressed in subpial astrocytes of the glia limitans [23–25]. In astrocyte endfeet, AQP4 anchors to the cell membrane by binding intracellularly with α-syntrophin, α-dystrophin, and dystrobrevin. Extracellularly, it binds to laminin and agrin, forming part of a protein scaffold called the dystrophin-associated protein complex (DAPC) [24, 26, 27]. This complex is especially relevant in the CNS, as it determines the polarization of AQP4 expression in perivascular astrocytes that form the BBB [28, 29]. Previous work of our group and others [30, 31], have shown that the expression of AQP4 in astrocytes changes during postnatal neurodevelopment of the CNS. In fact, unpolarized expression of AQP4 is found in astroglia cells, at early stages of astrogenesis, that help to populate white matter tracks [30]. And in mice, during the first postnatal week, AQP4 labelling is found exclusively on less mature glial cells in the CC, cerebellum, and spinal cord [31]. These astrocytes highly expressing AQP4 in the white matter at early maturation ages have been implicated in the very active myelination process that occurs at that age in the brain of newborn animals.

Then, the central objective of the present study was to examine how AQP4 deletion affects the development of the CC, with a focus on identifying whether critical processes such as oligodendrogenesis and myelination are impacted in AQP4-KO (AQP4-/-) animals. We focus on postnatal analysis, specifically on postnatal day 11 (P11), since that is the age at which the most important postnatal myelination phase of the CC begins, and also because at that stage AQP4 still has a relevant expression in this tissue. Shortly after that age, the expression of AQP4 in the CC of WT animals disappears and the signal for AQP4 expression moves toward more cortical regions, over the end-feet membrane of astrocytes surrounding cortical blood vessels [31]. Our results confirm that the absence of AQP4 in the CC of our experimental animal affects the normal transcriptomic and proteomic expression profile of this brain region. Additionally, we confirmed alterations in the myelination of axons in the CC of these animals. Verifying whether these CC alterations related to AQP4 expression absence cause atrophy of this tissue, which could be associated with postnatal neuropathology in these animals, is a promising task for future research.

## Materials and methods

### Animals care

Wild-type male and female mice (WT/C57BL/6) and AQP4-KO mice (AQP4^-/-^/ C57BL/6) were housed with a 12-hour light/dark cycle and ad libitum access to food and water. The AQP4^−/−^ mice were genotyped as indicated previously [32]. Mice were sacrificed under deep anaesthesia induced by a combination of 100 mg/kg ketamine (Pfizer, New York, NY, USA) and 10 mg/kg xylazine (Bayer, Leverkusen, Germany). All experiments were carried out according to the European Directive 2010/63/EU and the Spanish RD/53/2013 on the protection of animals used for scientific purposes. Animal procedures were approved by the Animal Research Committee of the Virgen del Rocío University Hospital (26/01/2017/017; University of Seville).

### RNA extraction and quantitative reverse transcription polymerase chain reaction analysis

The CC regions from the P11 animals were microdissected in ice-cold diethyl pyrocarbonate-PBS under a stereoscopic binocular microscope (Olympus SZX16, Tokyo, Japan). Total RNA was isolated using the RNeasy Micro Kit (Qiagen, Hilden, Germany) according to the manufacturer’s instructions. The cDNA synthesis was performed using the QuantiTect reverse transcription kit (Qiagen), and relative mRNA expression levels were measured using quantitative real-time polymerase chain reaction (RT-qPCR) with a ViiA 7 real-time PCR system (Thermo Fisher, Waltham, MA, USA). RNA expression levels were normalised (using 18 S ribosomal mRNA), and all samples were analysed in triplicate. Primer Express software v2.0 (Applied Biosystems, Waltham, MA, USA) was used to design all primers used (Table 1). RNA and cDNA quality and quantity were assessed with a NanoDrop ND-1000 UV–vis spectrophotometer (Thermo Fisher).

**Table 1:** Primers for qPCR

Gen Symbol	Gen	Forward	Reverse
Aqp4	Aquaporin 4	CCGTCTTCTACATCATTGCACAGT	GCGGTGAGGTTTCCATGAA
Aspa	Aspartoacylase	CCCTGCTCTGTTTATCTCATTGAG	TGAGGCTGAGGACCAACTTCTAT
ApoE	Apolipoprotein E	GGCCCAGGAGAATCAATGAGTA	GGCCAGAGAGGTGCTTGAGA
Cd44	CD44 Molecule (IN Blood Group)	AGTCACAGACCTACCCAATTCCTT	GGTGTGTTCTATACTCGCCCTTCT
Cldn11	Claudin 11	TGCCGAAAAATGGACGAACT	ACGGAGGCAGCAATCATGA
Cspg4	Chondroitin Sulfate Proteoglycan 4	CTGCAGAAGGAGGATCATTCTCA	GTCTCACTTCCATCATGCACGTA
Gfap	Glial Fibrillary Acidic Protein	TGGACACCAAATCCGTGTCA	ACGTCCTTGTGCTCCTGCTT
Gja1	Gap Junction Protein Alpha 1	CAACCCGGTTGTGAAAATGTCT	AGCTTCTCTTCCTTTCTCATCACATAG
Mal	Mal, T Cell Differentiation Protein	TCTAGAGCAAAGTGAGAGGCAATG	CTTTGCAGATTCTCCATGTCCTT
Mbp	Myelin Basic Protein	ACACACGAGAACTACCCATTATGG	GTTCGAGGTGTCACAATGTTCTTG
Mog	Myelin Oligodendrocyte Glycoprotein	GAGAGGAAAACTTCGTGCAGAAG	CAAGAACAGGCACAATAACAAACAG
Olig2	Oligodendrocyte Transcription Factor 2	CAGCATCCACTCCGTGTCAGT	GCGTTGTGGCCGTTTTGTAC
Opalin	Oligodendrocytic Myelin Paranodal and Inner Loop Protein	TGCAGTGCCAGTGAGAGAGAA	GACAACTGGAGAGGATGTCGTATTC
Pdgfra	Platelet Derived Growth Factor Receptor Alpha	AGACGGATGAGAGTGAGATCGAA	TCATCCCGAGAGGCACAAA
Sox10	SRY-Box Transcription Factor 10	ACCCTCACCTCCACAATGCT	GCCTCTCAGCCTCCTCAATG

### Microarray analysis

For the transcriptomic analysis of tissue samples, the ClariomS^TM^Micro Assay mouse kit (Thermo Scientific; Affymetrix) was used. A total of 3 samples per condition were included in the study (each one being a pool of 3 independent biological samples). The integrity of the RNA was evaluated by capillary electrophoresis with the 2100 bionalyzer (Agilent). The preamplification of the extracted RNA was carried out using the GeneChip™ IVT Pico kit (Thermo Scientific) and according to the protocol standardized by the manufacturer. Next, 5 µg of the resulting cDNA was fragmented and labelled for hybridisation in the ClariomTM Mouse Assay (Thermo Scientific). This was followed by the washing, labelling (GeneChip™ Fluidics Station 450; ThermoScientific) and scanning (GeneChip™ Scanner 3000; Thermo Scientific) steps, which were performed according to the provided protocols.

The data were preprocessed (with background correction, normalisation, and summarization) using the RMA (Robust Multichip Average) method, using the BrainArray annotation library pd.clariomsmouse.mm.entrezg (version 25.0.0) for mapping probes to genes. Differential expression analysis was performed with the limma package (version 3.46.0) [33]. To extract biological information from the list of differentially expressed genes, gene set enrichment analysis (GSEA) was performed [34]. The gene sets used come from the Signature Database (v7.4). Finally, through the TAC software (Transcriptome Analysis Console; Affymetrix, Thermo Scientific) we obtained the graphs of the Volcano-type analysis and the heatmap shown in Fig. 3.

### Electron microscopy

For transmission electronic microscopy (TEM), mice were perfused transcardially with 2.5% glutaraldehyde in phosphate or cacodylate bufer (0.1 M, pH 7.5), and the brains were postfxed for 2 h in the same fixative. A dissection of the anatomically region of interest (corpus callosum, + 0.62 to − 0.10 mm rostro-caudal relative to Bregma), was performed after vibratome cutting. Subsequently, these samples were rinsed, post-fxed in 2% osmium tetroxide, and embedded in epoxy resin. Ultrathin sections from the samples were obtained and examined with an electron microscope (Zeiss Libra 120).

### Measurements of the degree of myelination

To study the level of myelination of the axons of the corpus callosum, the semi-automatic free application MyelTracer was used [35]. In this system, images of the corpus callosum axons acquired at 4000 magnifications (> 20 per animal) were introduced and after introducing the scale, the developers’ instructions were followed. This procedure consisted of making a freehand trace of the myelinated layer on its internal side, in contact with the axonal cytoplasm, as well as on the external side. This information is interpreted and optimized by the program, which returns relevant values ​​such as the axonal diameter or the g-ratio value. The latter is the broadest and most traditionally used parameter to describe the degree of myelin enrichment of the axon [36], and its value, between 0 and 1, is inversely proportional to the degree of myelination of the axon. Below, in the section of immunofluorescence, a short sentence was included to briefly describe a staining, similar to the DAPI procedure that was used in few experiments to evidence the presence of myelin in CC regions in a less quantitative way.

### Evaluation of water content

To determine the water content in the brain, approaches described by different authors were followed [37–39]. After the animal was sacrificed, the brain was removed and weighed immediately. Afterwards, the organ was placed in a 65 °C incubator and left to dry for 48 h. After this time, the brains were weighed again, and the percentage of water was determined by the weight difference between the initial weight and the end of the drying process. This method was followed to evaluate the percentage of water in WT animals of 7, 11, 20 and 60 days.

### Immunofluorescence staining

All immunofluorescence and electron microscopy analyses were performed on coronal brain sections that included rostral regions of the corpus callosum (+ 0.62 to − 0.10 mm rostro-caudal relative to Bregma). Coronal brain Sect. (30-µm thick) were cut in a cryostat (Leica) and mounted on Superfrost Plus slides (Thermos Scientific). The IF protocol were previously described [31, 40] and briefly summarized as follow. The slices were washed twice for 5 min in 0.1 M PBS, then permeabilized with the application of PBT-0.3% [0.1 M PBS with 0.3% (v/v) Triton (Sigma)] and washed with PBT-0.1% on two occasions during 5 min before the blocking step. This step was carried out in 200 µl of blocking solution [10% goat or horse serum (Sigma) and 1 mg/ml bovine serum albumin (BSA, Sigma), in PBT-0.1%], using incubation chambers (Electron Microscopy Sciences). After 1 h, the solution was removed and the different primary antibodies used were dissolved in blocking solution at the specific concentrations and left for incubation overnight at 4 °C. The primary antibodies used in this work were: anti-AQP4 (1:100; Alpha Diagnostic, Int), anti GFAP (1:300, Sigma), anti CD11c (1:500, Biorad), anti APC (CC1) (1:100, Millipore), anti Olig2 (1:100, Sigma) and anti PDGFRa (1:200, R&D systems). Anti-mouse IgG conjugated with Alexa Fluor488 (1:400, Invitrogen), anti-rabbit IgG conjugated with Cyanine CyTM3 (1:200, Jackson Immunoresearch), anti-goat IgG conjugated with Cyanine CyTM3 (1:200, Jackson Immunoresearch) and anti-mouse conjugated with Alexa Flour653 (1:400, Invitrogen) were used as secondary antibodies, for two hours in the dark. The nuclei were stained with DAPI (1:1000, Sigma); and in some occasions, the Fluoromyelin^TM^488 (Invitrogen, Walthman, MS, EE.U.) reagent was added to the DAPI staining solution at 1:200 dilution ratio, to evaluate the presence of myelin. Incubation for 10 min was prolonged in these cases. Then, sections were mounted with Dako fluorescence mounting medium (Dako). Confocal images were acquired using a Leica Stellaris 8 confocal microscope. In all cases, defined areas were established in the histological section and several layers were acquired reading on the Z axis (Z-stack), defining a distance of 0.7 μm between the layer and layer. The images obtained were evaluated with FIJI [41] and in certain cases three-dimensional reconstructions of the obtained markings were performed using Imaris Software (Bitplane, Belfast, United Kingdom) for this purpose.

### Statistical analysis

The data are presented as mean ± standard error of the mean (SEM), and the statistical test performed is indicated in each figure legend. For all the analyses performed, normality was checked using the D’Agostino and Pearson test or the Shapiro–Wilk test. When this was confirmed, a variance analysis was performed with Tukey’s HSD *post hoc* analysis for multiple group comparisons or Student’s t-test (for 2-group comparisons); otherwise, the nonparametric Kruskal–Wallis H test (for multiple comparisons) or the Mann–Whitney U test (for 2-group comparisons) was used. GraphPad Prism Software (8.4.2 version, San Diego, CA, USA) was employed for the statistical analysis and graph design.

## Results

### Expression of genes associated with oligodendrogenesis in the corpus callosum and its correlation with AQP4 expression

**Fig. 1:**
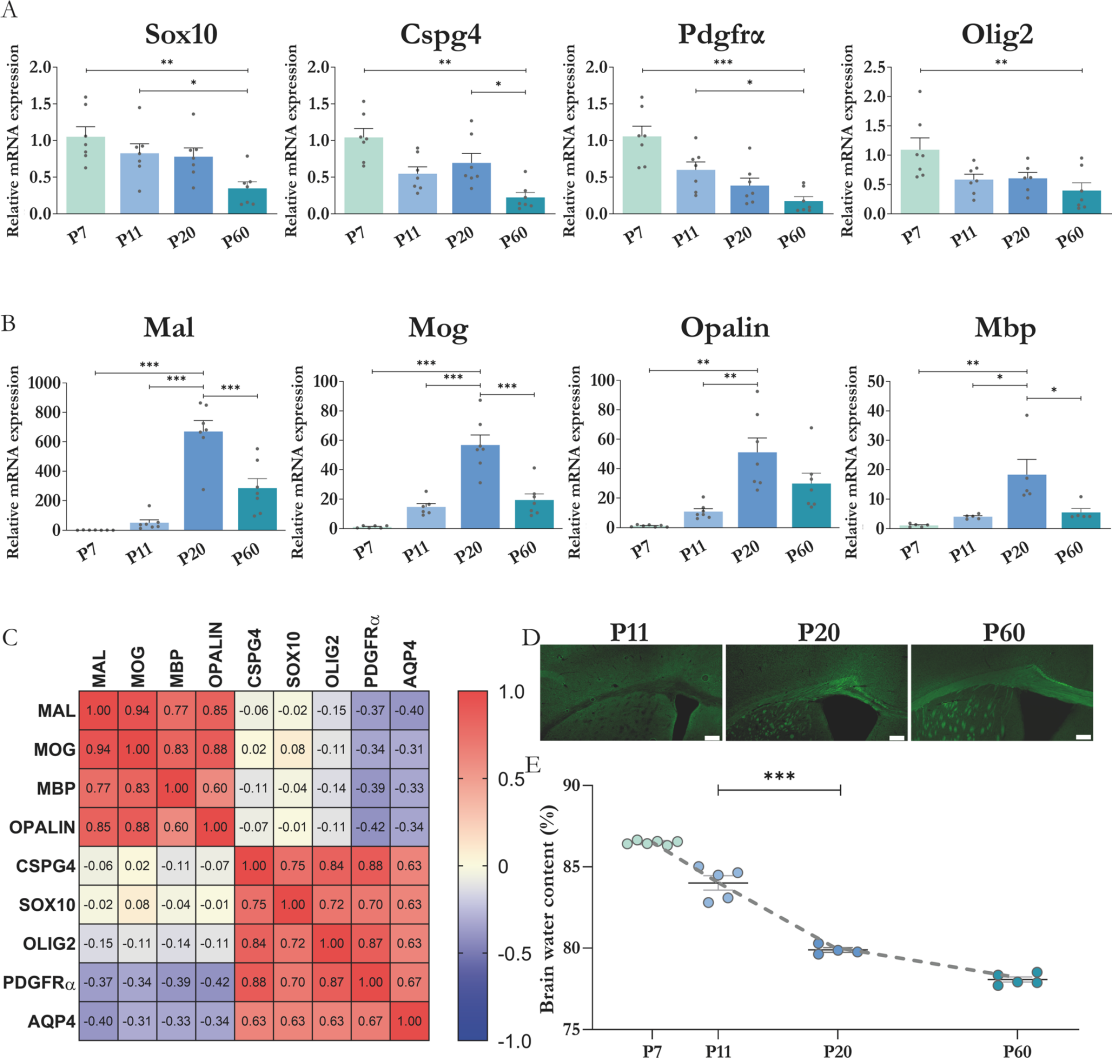
Expression levels of genes associated with oligodendrocytes, OPCs populations and myelin formation, and correlative analysis with AQP4 expression. Changes in relative mRNA levels for genes of OPCs (**A**) and mature oligodendrocytes (**B**). *N* = 5–7 per group. (**C**) Matrix of correlations by Pearson’s coefficient between the relative levels of the assessed markers and the levels of the evaluated markers and AQP4. Time course for levels of mRNA and protein of AQP4 were previously shown [31]. (**D**) Histological assay against myelin (Fluoromyelin-AF488 compound) from stage P11 (onset of myelination) to adult tissue. Scale bar = 150 μm. (**E**) Quantification of water percentage in brains of the different age groups. *N* = 3–6 per group. Significant differences between groups were evaluated by one-way ANOVA followed by Tukey’s post hoc test (*p < 0.05; **p < 0.01; ***p < 0,001)

In a previous work [31] we confirmed a transient expression of AQP4 in CC, that peak at maximum levels between P11-P20, just when the gross myelination process of CC occurs. Here, we wanted to relate the expression of AQP4, with the expression profile of genes characteristic for OPCs, such as *Sox10*,* Pdgfrα*, *Olig2* and *Cspg4*, and with that of genes distinctive of mature OL, such as *Mbp*, *Mog*,* Opalin* and *Mal*; analysing corpus callosum samples from mice between P7 and P60. As shown in Fig. 1, the changes in the expression of the 4 markers associated with OPCs (*Sox10*,* Pdgfrα*,* Olig2 and Cspg4*) followed a similar dynamic (Fig. 1A), with maximum expressions levels reached at the early postnatal point P7 with respect to levels detected in the adult stage (P60). Reductions, between 70% for *Olig2*, and close to 90% for *Pdgfrα*, were observed with respect to the levels obtained at P7 (*p* < 0.01). For another side, genes encoding proteins associated with mature oligodendrocytes (*Mbp*,* Mog*,* Opalin*, and *Mal*) reached their maximum expression at the stage P20 (Fig. 1B), indicating the highest rate of myelination at that point. In adult tissue (P60), these genes maintain expression levels significantly higher than the expression levels in earlier postnatal ages (P7 and P11), although lower than the levels detected in P20. The expression of markers for OPC and OL (Fig. 1A and B, respectively) was compared with the expression levels of AQP4 determined in these same tissues in a previous work [31], and such correlation analysis, at different ages, is shown in Fig. 1C. The analysis, carried out as a Pearson parametric correlation, showed that the degree of expression of AQP4 associated significantly and directly with that of specific genes for OPC, and by contrast, an inverse relationship, although more modest, was observed between the levels of AQP4 and that of mature OL genes. (Fig. 1C). Finally, AQP4 levels were correlated with tissue myelination levels. Using histological staining with the compound Fluoromyelin^TM^488, myelin was stained and the sudden enrichment in myelin levels was verified between stages P11 and P20 (Fig. 1D), with a small increase toward P60. These results clearly contrasted with those of the water content in brain tissue, whose determination highlighted the greatest significant decrease between P11 and P20 (greater than 4 points in total percentage values, *p* < 0.001). The importance of AQP4 in the process of water evacuation in the brain during the postnatal stage could play a greater relevance in the myelin maturation process, given the hydrophobic nature of this cellular layer.

### Identification of the cellular origin of AQP4 labeling in the corpus callosum

**Fig. 2:**
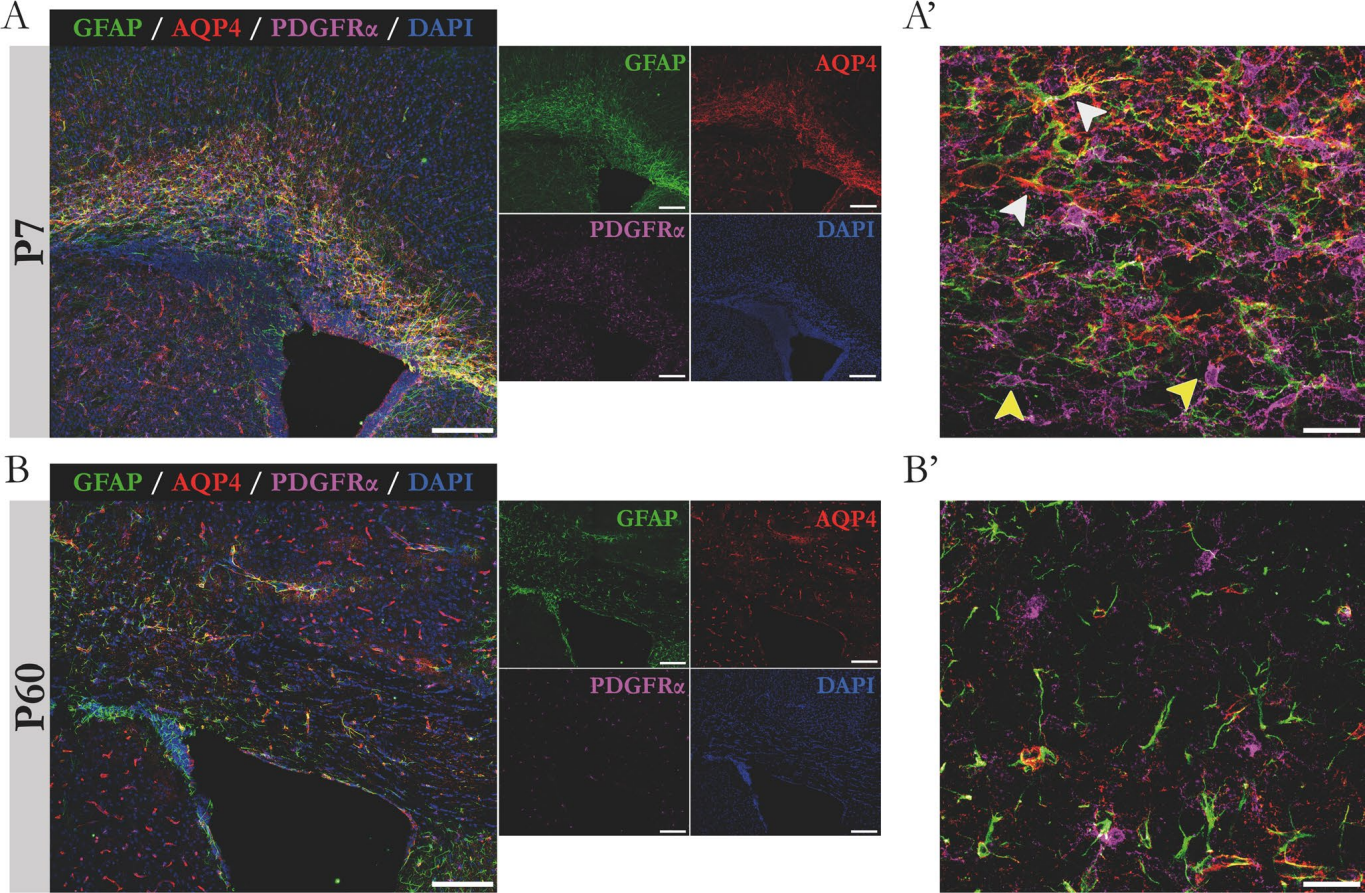
Immunolabeling of astrocyte and OPCs in the corpus callosum of animals at P7 and P60. Comparative micrographs of the expression of astrocyte markers (GFAP) and OPCs (PDGFRα) in combination with AQP4 signal in animals at P7 (A) and P60 (B). Magnified images (A‘ and B’) reflect the increased presence of astrocytes (white arrowheads) and OPCs (yellow arrowheads) in postnatal developing tissue. Note the increased signal for AQP4 around the astroglial cell type. Scale bars = 150 μm (A and B) and 15 μm (A‘ and B’).

Cellular processes such as oligodendrogenesis and astrogenesis are actively occurring in the corpus callosum at postnatal age seven (P7). To know which type of cell is responsible for the high expression of AQP4 at that age, immunofluorescence assays were performed, using GFAP y PDGFRα as markers for astrocytes and OPCs, respectively. In Fig. 2, representative images of the immunoassays are shown and the results of the comparison between animals in the postnatal stage P7 (Fig. 2A) and young adults P60 (Fig. 2B) are included. As a glancing view, a greater presence of both cell types, astrocytes and OPCs, is appreciated in the corpus callosum of P7 animals, compared to what was obtained at P60, where both cell types were drastically reduced; in line with what has already been described for AQP4 (Fig. 1C and previous work [31]). Images at higher magnifications allowed us to visualize that AQP4 labelling overlapped with GFAP (white arrows), and not with PDGFR (yellow arrows), used as a oligodendrocyte progenitor labelling marker. These results confirmed that astrocytes are the cells responsible for AQP4 expression and therefore, the greater abundance of astrocytes in the tissue during the postnatal period would be responsible for the greater prominence identified at that age for the two glial markers analyzed, GFAP and AQP4.

### A comparative transcriptomic analysis of the postnatal corpus callosum in WT versus KO mice

**Fig. 3:**
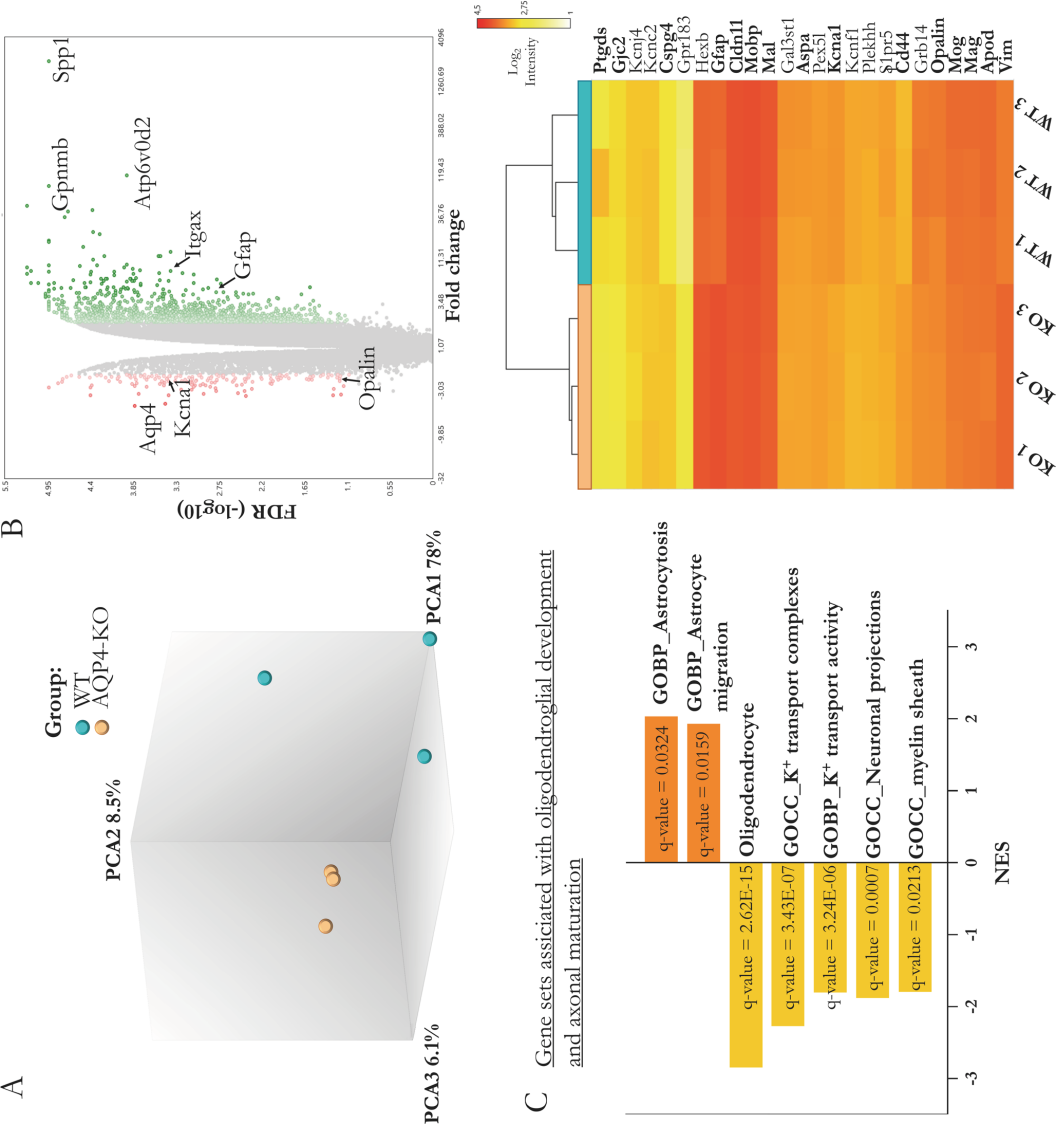
Comparative transcriptomic analysis (Microarray) of the corpus callosum of WT and AQP4-KO mice at P11 (A) Component analysis (PCA) of the gene profile of the samples included in the study by condition. (B) Volcano plot detailing the 4953 genes significantly expressed by FDR/q value <0.05 (in green gradient according to the degree of overexpression or red gradient for those identified as inhibited). The graphs reflect the gene sets (GS) associated with astrocytes, oligodendrocyte and myelin development (C) that showed significant differential expression (q value<0.05). On the right-hand side of each, heatmaps with highlighted GS genes are shown (those that were analysed by RT-qPCR are indicated in bold).

To better understand the relevance that AQP4 expression has on the entire transcriptomic profile of the corpus callosum, we decided to perform a Microarray analysis (Affymetrix), comparing total transcriptomic profiles of corpus callosum samples from WT and AQP4-KO mice at the age of P11. The global screening results showed an important homogeneity between groups in the different samples (Fig. 3A), which would allow us a greater grouping of the gene profiles and therefore a higher level of gene expression differences between conditions. In fact, as a consequence of this good delimitation of gene profiles, the differential expression study showed significant changes according to the predefined statistical thresholds (FDR < 0.05) with a FC > 1.5 in 4953 genes (840 down-regulated and 4113 up-regulated), in the comparison of AQP4-KO versus WT as represented in the volcano plot (Fig. 3B). As shown in Fig. 3C, the analysis showed that the tissue from the KO mice has extensive enrichment in the gene profile of the astrocytes populations and genes associated to astrocytes migration (normalised enrichment score [NES] of about 2.0 in both cases). Overexpression of genes such as *Gfap*,* Vim*, or *Cd44*, typically associated with astrocytes, was observed and highlighted with significant increases in the KO mice. Also, clearly overexpressed in the KO mice were genes expressed by the microglial CD11c subtype, such as *Spp1*,* Gpnmb*,* Atp6v0d2* and *Itgax*, as highlighted in the Volcano plot (Fig. 3B). By contrast, a decrease in gene enrichment of gene sets (GS) associated with the oligodendrocyte population (NES of -3.00) was observed in the AQP4^−/−^ mice. And there was also a decrease in the expression levels of genes grouped under ontological terms (GO) of biological processes related to formation of myelin sheaths, neuronal growth or genes associated with K^+^ ion channels and transporters (NES around of -2.00). In the heat map shown on the right side of Fig. 3C, significant genes related to astrocytes and oligodendrocytes populations are indicated, and those genes that were validated by RT-qPCR are in bold.

### Study of alterations associated with the astrocyte lineage

**Fig. 4:**
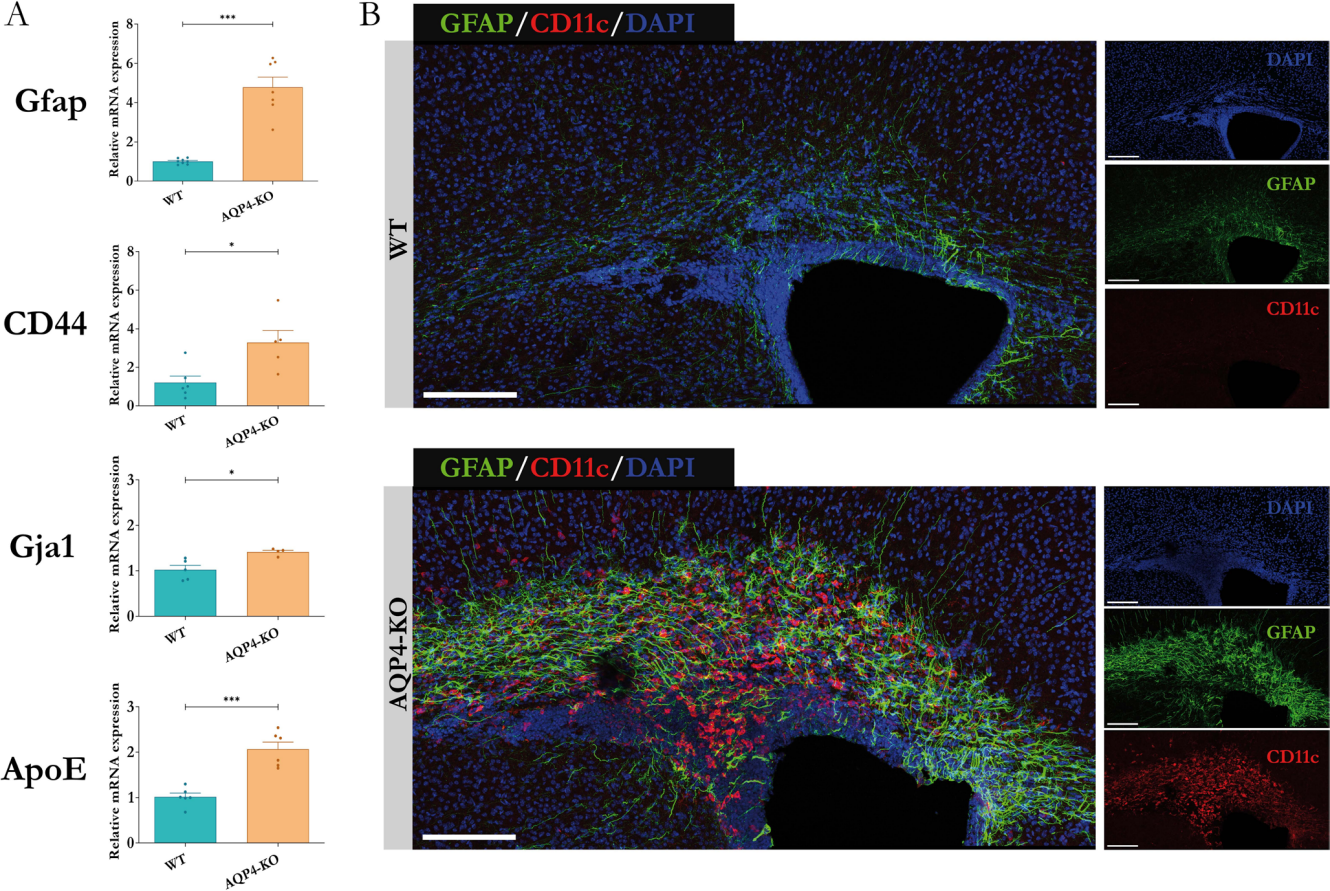
Comparative analysis of astrocyte abundance in the corpus callosum during development of WT and AQP4-KO mice at P11. (A) Evaluation of gene expression associated to astrocyte function. (B) Immunofluorescence labelling of astrocytes (GFAP+) and CD11c+-microglial cells in the corpus callosum at P11 (WT vs AQP4-KO animal). *N = 4-7* per group. Significant differences between groups were assessed by one-way ANOVA followed by Tukey's post hoc test (*p<0.05; ***p<0,001).

Based on the results from the transcriptomic analysis, we performed qRT-PCR assays to validate the gene expression changes of markers associated with astrocytes, such as *Gfap*,* Cd44*,* Gja1* y *Apoe* [42–44], and the results are shown in Fig. 4A. Statistically significant values (*p* ≤ 0,05) were obtained in all genes, finding a significant overexpression of 4.19 folds for *Gfap*, 3.28 folds for *Cd44*, 1.42 folds for *Gja1* and an increase of 2.01 folds for *Apoe*, in the samples from AQP4-KO animals compared to WT animals. To label astrocyte populations in CC by immunofluorescence, the antibody against GFAP was used (Fig. 4B). GFAP labeling is observed in the CC of both WT and AQP4-KO animals. However, stronger GFAP signal, indicative of a greater expansion of astrocytes, is observed throughout the CC in the AQP4-KO than in the WT, which corroborates the hypothesis about the existence of astrocyte activation or astrocytosis in the AQP4-KO mouse at P11. Additionally, the staining with CD11c antibody, to label that specific type of microglia, confirmed the expression of this marker only in the CC of the AQP4-KO mouse and not in the CC of WT P11 animals as indicated previously [21].

## Study of alterations associated with the oligodendrocyte lineage

### Evaluation of OPCs

The appearance of mature myelinating oligodendrocytes constitutes the last step in the cellular differentiation of the oligodendrocyte lineage, which begins with the appearance of OPCs from neural progenitor cells [45]. Given the reduction in the presence of oligodendrocytes observed in the CC of the AQP4-KO animal at P11, we wondered if the abundance of OPCs could also be affected. For its study, we first performed qPCR assays to evaluate the degree of gene expression of markers of these cells, and then, IF assays against the markers PDGFRα and OLIG2, which, when combined, allow us to identify the population of OPCs [46].

**Fig. 5:**
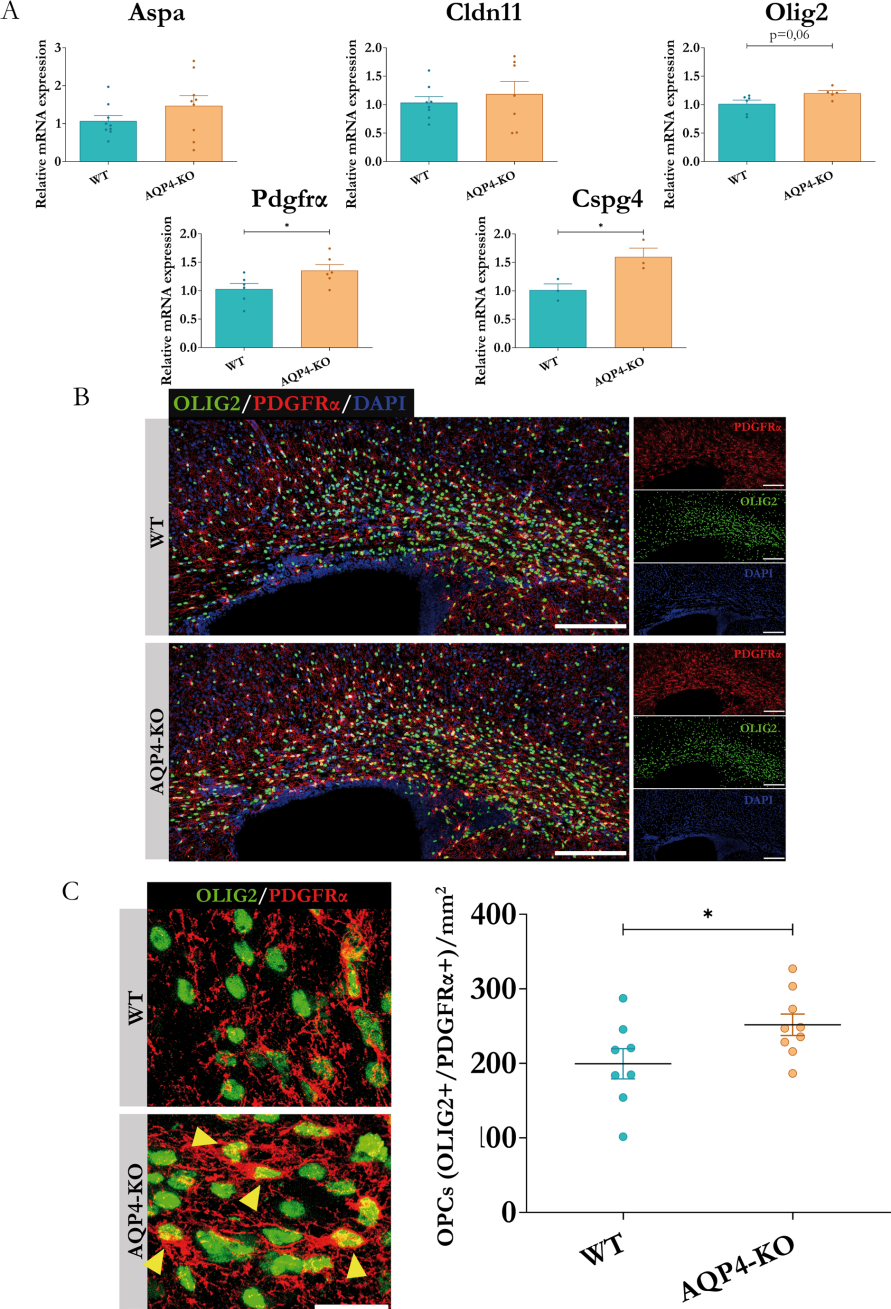
Differential expression of genes associated with OPCs and quantification of their cellular abundance by immunolabelling in the corpus callosum (A).RT-qPCR validation of expression levels in the corpus callosum (P11) for genes associated with undifferentiated OPCs. N=3-8 per condition. (B) Images of immunofluorescence assays to assess tissue enrichment of OPCs (PDGFRα+/OLIG2+ cells) on coronal brain slices in the two study conditions. To the right of each image, the signals obtained for both markers and the nuclear staining agent DAPI are represented. (C) Enlarged micrographs of the regions where PDGFRα+/OLIG2+ cells were quantified (yellow arrows) and quantification performed with the mean values per animal of the number of cells per unit area. *N=8-9* per group. Unpaired Student's t-test for significance analysis. *p<0,05. Scale bars = 150 μm (B) or 15 μm (C).

As a result of the qPCR experiments (Fig. 5A) we obtained significant increases in the expression levels of genes characteristic of OPCs, such as *Cspg4* or *Pdgfrα* in the CC of AQP4-KO mice. In line with this, the evaluation by IFs of the OPC markers, PDGFRα and OLIG2 (shown in Fig. 5B), demonstrated a significant increase (greater than 20%) in the presence of OPCs in the tissue of the AQP4-KO animals (Fig. 5C). These results suggest a delay in the maturation of the oligodendrocyte lineage in the CC of AQP4-KO animals, which occurs concurrently with activation of astrocytes and microglia, although a direct causal relationship between these events cannot be established from the present data. To further clarify whether the total number of cells of the oligodendrocyte lineage was affected, we quantified all OLIG2 + cells in the corpus callosum, including both OPCs and mature oligodendrocytes. The analysis revealed no significant differences between WT and AQP4-KO animals (WT: 361 ± 25 vs. KO: 355 ± 22 cells/mm^2^, *p* = 0.51; (data shown in Supplementary figure S1), indicating that the total number of OLIG2 + cells was preserved in the absence of AQP4. Therefore, the observed changes in Fig. 5 correspond to an altered balance between OPCs (OLIG2+/PDGFRα+) and mature oligodendrocytes (OLIG2+/CC1+), rather than a reduction in the overall number of oligodendroglial lineage cells.

### Evaluation of mature Oligodendrocytes

**Fig. 6:**
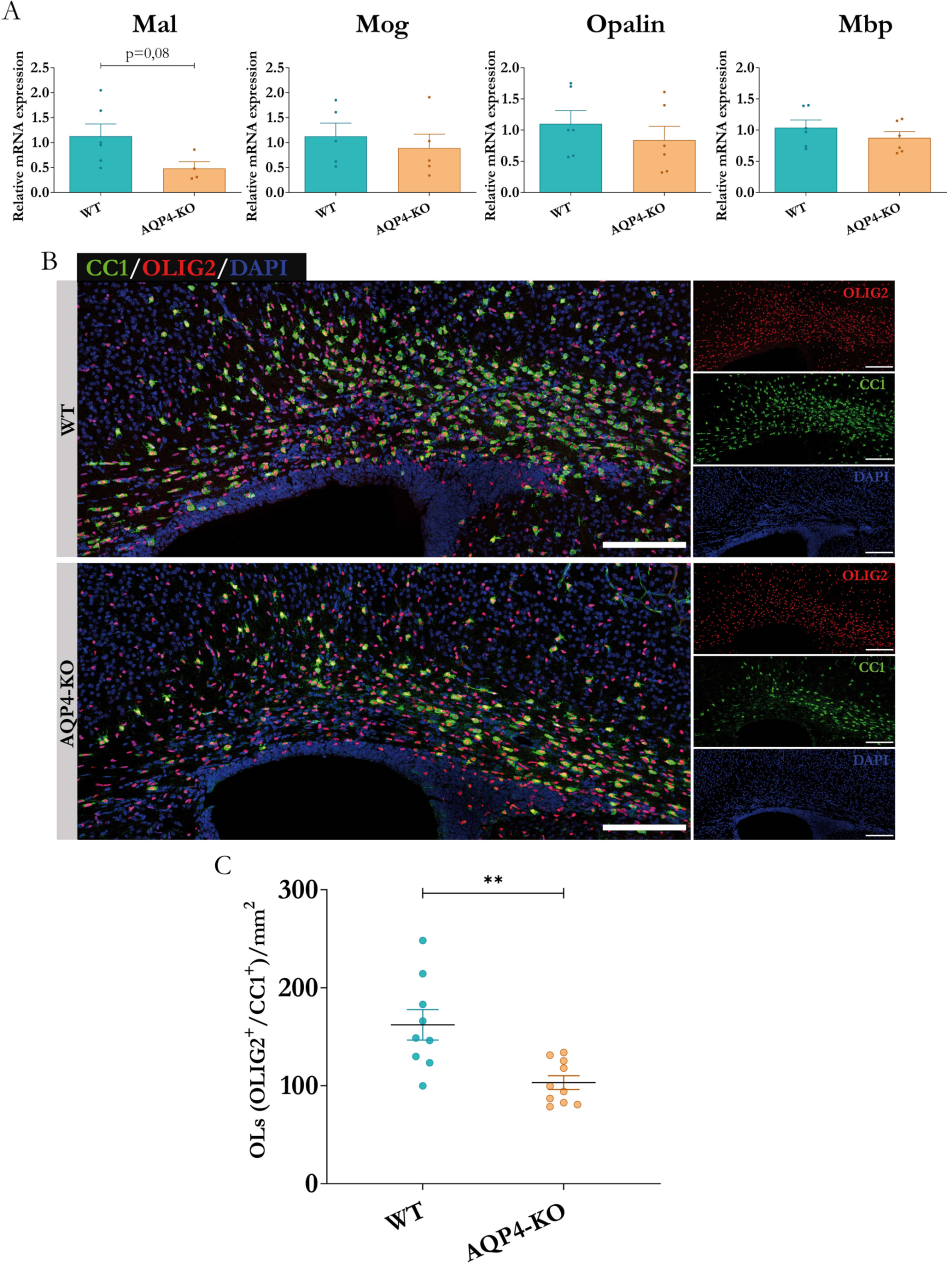
Comparative study of gene expression associated with mature oligodendrocytes and quantification of their cellular abundance by immunolabelling in the corpus callosum. (**A**) RT-qPCR validations of expression levels in corpus callosum (P11) for genes associated with the differentiated oligodendrocyte cell type. *N* = 3–8 per condition. (**B**) Immunofluorescence assays to assess tissue enrichment of oligodendrocytes (CC1+/OLIG2 + cells) in the two study conditions. To the right of each image, the signals obtained for both markers and the nuclear staining agent DAPI are depicted. Scale bars = 150 μm. (**C**) Quantification of the number of oligodendrocytes per area and comparison between the two conditions. *N* = 9–10 per group. Unpaired Student’s t-test for significance analysis. *p < 0.05, **p < 0.01

Based on the data from the transcriptomic analysis, we performed qRT-PCR assays to validate the gene expression changes of the markers associated with mature oligodendrocytes. To do this, we explored the expression levels of several representative genes of mature oligodendrocytes and its myelinogenic function such as: *Mal*,* Mog*,* Opalin*,* and Mbp* [47, 48] in biological samples independent of those used for the microarray, coming from CC of WT and AQP4-KO animals. The results obtained (Fig. 6A) indicated the absence of significant changes in the levels of gene expression between groups, although trends towards reduction were observed, especially in the *Mal* gene (*p* = 0.08, 48% reduction in the level of expression). Despite this lack of changes in the levels of gene expression, we quantified the abundance of oligodendrocytes in the tissue, using immunohistochemical assays against OLIG2 and CC1, two marker proteins that, when colocalized, allow us to identify distinctively differentiated oligodendrocytes. The result of this analysis (Fig. 6B) reflected a greater abundance of CC1 positive cells in the CC of the WT animals, with an ostensibly lower density in the tissue of the transgenic animal. The quantification carried out from the images (Fig. 6C) revealed a 37% drop in the OL population count (OLIG2+/CC1+)/mm2 in the CC of the AQP4-KO animal.

## Study of alterations in the myelination

**Fig. 7:**
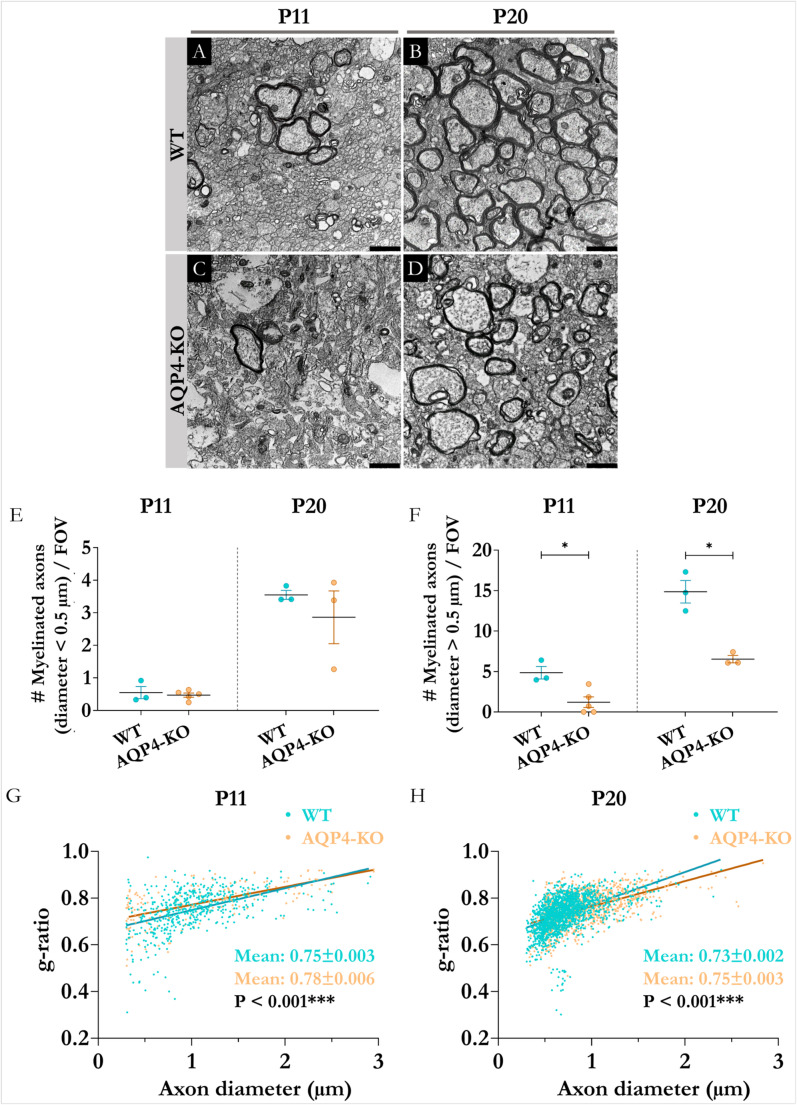
Study of the degree of myelination of corpus callosum axons in postnatal WT and AQP4-KO animals. Electron micrographs of the corpus callosum of WT and AQP4-KO animals at P11 and P20 (A-D). Quantification by field of view (FOV) of the number of myelinated axons of smaller (<0.5 μm, E) or larger (>0.5 μm, F) diameter. Quantification of myelin sheath thickness (g-ratio) in animals at P11 (G) and P20 (H) of study conditions. *N=3* per group. Unpaired Student's t-test for significance analysis. *p<0,05. Scale bars = 1 μm.

Finally, we were interested in determining whether these gene and protein changes indicative of alterations in the proportions of oligodendrocyte lineage cells could be impacting myelination. To do this, we evaluated by transmission electron microscopy (TEM) the state of myelination in the CC at P11, of both types of animals. Given that myelination at P11 is still incipient, we extended the comparative analysis also to point P20, where we had previously shown the existence of a higher degree of expression of mature oligodendrocyte genes in the CC of the WT animal (Fig. 6). Micrographs, acquired by TEM, of the myelination state of the tissue (Figs. 7 A-D) showed lower levels of myelination in the axonal fibers of the CC of the transgenic animal compared to the WT. Indeed, quantification of the number of myelinated axons per field of view demonstrated significant drops in the number of larger caliber axons (> 0.5 μm) at P11 (Fig. 7F); while no changes were observed for smaller caliber axons (Fig. 7E). Finally, the assessment of the degree of myelination through the g-ratio (parameter inversely proportional to the thickness of the myelin), indicated a decrease in the amount of myelin in the tissue from the AQP4-KO animals both at P11 (Fig. 7G) as at P20 (Fig. 7H), with respect to the WT of each point. As a whole, the results obtained for CC a in this section highlighted a lower maturation state in the CC of transgenic AQP4-KO animals. To rule out that the observed alterations in myelination were secondary to changes in axonal structure, we quantified both the total number and diameter of axons in the corpus callosum of WT and AQP4-KO mice at P11 and P20. No significant differences were detected between genotypes in either parameter, indicating that axonal density and caliber remain unaffected by AQP4 deficiency. These findings suggest that the myelination delay observed in AQP4-KO animals is not attributable to intrinsic axonal alterations (Supplementary Figure S2).

## Discussion

The complexity of myelination during development involves not only the formation of myelin sheaths around axons, but also the implementation of a precise transcriptional program in undifferentiated OPCs that leads to the generation of mature oligodendrocytes, which are directly responsible for this task. After observing changes in AQP4 expression in tissues where myelination occurs, such as the CC, cerebellum, and spinal cord [21, 31], we investigated whether constitutively eliminating AQP4 expression in animals would alter the expression of postnatal myelination marker genes in the CC. First, we determined the gene expression profile of markers related to the oligodendrocyte differentiation process in the CC of WT animals at different ages. Then, we analyzed the levels of representative OPC and of mature OLs genes, comparing WT versus AQP4-KO animals. In WT animals, a close parallelism between the high expression of AQP4 and that of genes expressed by OPCs was observed at P7; whereas a progressive increase in the levels of OLs genes was observed from P7 to P20, corresponding with a progressively reduction of the expression of AQP4. These results indicate that AQP4 is required for the initiation of postnatal OPC differentiation; however, its expression appears to play a reduced role once myelination is fully established (Fig. 1).

The myelin staining analysis also showed a clear increase in myelin content as one progresses from stage P11 to P20 and P60. Myelinization in the CC occurs in parallel with a reduction in tissue water content. Greater AQP4 expression coincides with a higher percentage of water in the brain and low myelin levels. Conversely, low AQP4 levels were found when myelin content is high, and the percentage of water is low. Such observation, already described by others [38, 49], allows us to suggest an interplay between water evacuation from the CC via AQP4, and the myelination of the brain tracts in this area: (i) developmental brain water content naturally decreases during maturation and this can coincide temporally with increasing myelin content; (ii) AQP4 expression in white-matter astrocytes during early postnatal stages likely contributes to water homeostasis that facilitates morphological and biochemical events required for OPC proliferation/migration (not just passive edema resolution); and (iii) while correlations do not prove causation, the combination of our transcriptomic, immunofluorescence and TEM data (reduced mature OLs and thinner myelin in AQP4-KO) supports a functional role for AQP4 in the developmental myelination program rather than a pure epiphenomenon.

The immunofluorescence assays, using antibodies against AQP4, GFAP, and PDGFRα, helped us confirm the astrocytic origin of AQP4 expression in CC. Overlapping of fluorescence signals was observed only when antibodies for GFAP and AQP4 were used in the CC at P7 and not P60, and was never detected when anti-AQP4 and anti-PDGFRα antibodies were used together (Fig. 2). Thus, we postulate that fibrous astrocytes GFAP+/AQP4+, abundantly present in the CC at P7, likely contribute to the initiation of OPCs differentiation, necessary to turn out into mature OLs, and could help to transport to them, lipids and all kinds of elements necessary in the synthesis of myelin [11, 14].

On the other hand, the comparative transcriptomic analysis (Fig. 3) revealed a clear decrease in genes associated with the mature OLs population and in genes related to the formation of myelin sheaths (*Mal*,* Mog*,* Opalin*, and *Mobp*) in the AQP4-KO mice. Conversely, enrichment in the gene profile of astrocytes (*Gfap*,* Vim* and *Cd44*), as well as in genes associated with astrocyte migration; and genes such as *Spp1*,* Gpnmb*,* Atp6v0d2*, and *Itgax* (Fig. 3B) which are expressed by CD11c+ microglial cells, as indicated previously [27], were significantly over expressed in the transgenic mice. It had been demonstrated that in the CC, the presence of a CD11c+ microglial cell type with high phagocytic capacity, helps the tissue maturation process through phagocytosis of both, the OPCs [11, 50, 51] and the recently formed OLs [8] that are produced in excess during the myelination process. Their ability to release factors with pro-myelination potential, such as Osteopontin [52], Galectin-3 [53] and especially Igf1 [51, 54], makes the CD11c+ microglial population fundamental to achieve normal levels of axon myelination in the CC and cerebellum, thereby promoting normal development of these tissues as previously noted [21].

The increased expression of *Gfap*,* Cd44*,* Gja1* and *Apoe*, that was observed in the AQP4-KO animal is also an indicator of the involvement of fibrous astrocytes (Gfap+/Cd44+), that together with the increased expression of CD11c+ microglia genes, will intend to favor the initiation of OPCs differentiation necessary to develop into mature OLs. Other elements released by astrocytes, such as *Pdgfα*,* Bdnf* and *Igf1* have been indicated, to be key in promoting the survival, migration and proliferation of OPCs [18–20]. Consistent with this, the GFAP-KO mouse shows lower amounts of myelin in the CC, optic nerve, and spinal cord at 6 months of age, which strongly supports the important role of astrocytes in correct brain myelination [40].

In our work, the glial fibrillary acidic protein (GFAP) was taken as the main marker that confirmed higher expression of fibrous astrocytes in the AQP4-KO mice (Fig. 4B). Also, the Cd44 gene, coding for the homonymous protein, was observed highly expressed in the CC of AQP4-KO animals. CD44, acts as a receptor for hyaluronic acid, an extracellular matrix component found in the brain that has been shown to be essential for the proper maturation of OPCs and effective brain myelination [11]. To provides trophic support to oligodendrocytes, astrocytes maintain intracellular communication through gap junctions. For that, they express Gja1, which encodes for connexin 43, the protein responsible for the formation of gap junctions [55]. Apoe, which encodes for apolipoprotein E, a protein involved in fat metabolism, that improves the correct passage of cholesterol to the oligodendrocytes [44], was also overexpressed in the transgenic mice. Taking all these elements together, we propose that astrocyte proliferation and perhaps astrocyte overactivation, together with the abundant presence of CD11c+ microglia, increase the secretion of various trophic factors. These factors promote an increase in OPC production. However, OPC maturation to yield mature OLs fails for reasons that are still unknown reducing the myelination process. In the CC of the AQP4-KO mouse (Fig. 5), a significant increase in the abundance of OPCs, labelled as (OLIG2+/PDGFRα+), and genes associated to them as *Olig2*,* Pdgfra* and *Cspg4*, was detected by immunofluorescence staining, but for unidentified yet reasons the final transformation of OPCs into mature oligodendrocytes does not seem to be properly achieved. Thus, the number of mature oligodendrocytes, labelled as (OLIG2+/CC1+), was significantly (** *p* < 0.01) lower in the AQP4-KO animal than in the WT mice, as lowers were the levels of marker genes (*Mal*,* Mog*,* Opalin* and *Mbp*) for mature oligodendrocytes (Fig. 6). On line with this and consistently with a smaller number of mature OLs, a reduced number of myelinated axons was counted in the CC of AQP4-KO animals compared to the WT ones. The differences in the number of myelinated axons were especially significant for large axons, with diameter > 0,5 μm (* *p* < 0,05), either at P11 and P20. Moreover, the thickness of the myelin sheath in the myelinated axons, estimated as the g-ratio (Fig. 7), was thinner in the CC of AQP4-KO mice compared to that measured in the WT animals, evidencing a reduced capacity for myelination in the AQP4-KO animals.

Further specific analyses in adult animals will be required to confirm whether the myelination alterations detected at postnatal stages correspond to a developmental delay rather than a permanent defect. One possible explanation for our results comes from the recent work of Su et al. [19], who demonstrated that in the brains of mice, the formation of astrocyte endfeet on vessels correlates with the termination of OPCs perivascular migration and differentiation. The authors of this study showed that astrocyte endfeet physically displace OPCs from the vasculature by releasing sema-3a and 6a. These semaphorins bind to the OPCs, which terminates their migration over the vessel and displaces them away from the vessel, allowing subsequent differentiation of OPC into mature OLs. In our study, we did not evaluate how well the astrocyte endfeet attach to the brain vessels in transgenic animals. However, we hypothesized that astrocyte–vessel interactions are altered in AQP4-KO animals, resulting in reduced endfeet attachment and impaired displacement of OPCs from the vasculature. Supporting our hypothesis, we extracted the microarray for semaphorin family members and their receptors and included the results as Supplementary Table S1. Several semaphorins ligands and receptors [56, 57], display modest but significant expression changes in AQP4-KO versus WT animals (for example, *Plxnb2* and *Plxnc1* show upregulation, whereas *Sema4d* is downregulated, etc.; see Supplementary Table S1). Therefore, it is tentative to think that defective crosstalk between astrocytes and OPCs (Astrocyte endfeet → semaphorins → OPC displacement) ) is occurring in the AQP4-KO animal. This may hinder the normal differentiation of OPCs into mature oligodendrocytes as indicated by Su et al. [19], which would reduce the myelinization process in the CC of that animal. In demyelinating diseases such as multiple sclerosis, Sema3a, 3f, 6a, 4d, and 7a have been suggested to be upregulated in or around demyelinating lesions [57, 58]. These proteins may integrate with other signals to regulate the recruitment or differentiation of OPCs in remyelination of injured areas. Additionally, multiple semaphoring receptors have been identified in OPCs and with development-related expression patterns [56].

The connection between AQP4 and demyelinating diseases is well-known. One example is neuromyelitis optica (NMO), an autoimmune demyelinating disease that is characterized by the presence of anti-AQP4 antibodies in patients’ serum [59]. Although it has been suggested that the binding of these autoantibodies does not affect the channel’s water permeability [60], some studies propose that binding of these antibodies to AQP4 leads to channel internalization [61, 62] and astrocyte damage caused by massive immune activation. Moreover, anti-AQP4 antibody-mediated internalization (or other mechanisms that damage astrocyte endfeet) may precipitate BBB breakdown via complement or immune processes, and such astrocyte injury may in turn affect oligodendrocyte viability and myelination [63]. The suppression of metabolic support from astrocytes to oligodendroglial cells is proposed as a mechanism that causes degeneration of these cells in NMO. Similarly, in other early-manifesting diseases with severe hypomyelination, such as leukodystrophies [55], causes of genetic origin, which often affect genes characteristic of astrocytes, are central to the disease’s origin. Alexander disease, one of the most common types of leukodystrophies, arises from mutations in the gene that codes for GFAP. Its predominant expression in the fibrous astrocytes of the white matter plays a key role in forming intermediate filaments of the cytoskeleton, which are essential for anchoring membrane complexes such as gap junctions and GLT-1 glutamate channels. These complexes play a crucial role in the development of oligodendrocytes and myelinogenesis. Interestingly, imbalances in AQP4 expression have also been identified as a characteristic feature of this and other leukodystrophies [64, 65], which suggests a potential role for AQP4 in the pathophysiology of these diseases.

In conclusion, our results provide evidence implicating AQP4 in the cell activation of astrocytes and microglia necessary to initiate the maturation process of oligodendrocyte precursors. The congenital absence of AQP4 in the CC of the AQP4-KO animal, result in a reduced number of mature oligodendrocytes and therefore a reduced capacity to achieve myelinization of CC axons. We propose that congenital alterations in the *AQP4* gene expression, affecting the postnatal development of the CC, could potentially explain, at least in part, pathologies associated with myelination problems at some point in life.

## Supplementary information

Below is the link to the electronic supplementary material.

Supplementary File 1 (DOCX 60.0 KB)

Supplementary File 2 (PDF 44.3 KB) Quantification of total OLIG2⁺ cells in the corpus callosum of WT and AQP4-KO mice at P11. Graph showing the mean ± SEM of the total number of OLIG2⁺ cells (including OPCs and mature oligodendrocytes) per mm² in the corpus callosum of WT and AQP4-KO mice at P11. No significant differences were found between genotypes (WT: 361 ± 18 vs KO: 355 ± 21 cells per mm²; n = 8 animals per group; unpaired Student’s t-test).

Supplementary File 3 (PDF 35.5 KB) Quantification of axonal diameter in the corpus callosum of WT and AQP4-KO mice. Plots showing the mean ± SEM for total axonal diameters at P11 and P20. No significant differences were detected between WT and AQP4-KO mice in either age analyzed (P11 and P20), indicating that axonal size remain unaltered in the absence of AQP4. No significant differences were found between genotypes (WT vs KO, n = 3-5 animals per group; unpaired Student’s t-test).

## Data Availability

https://idus.us.es/browse? type=author&value=Echevarr%C3%ADa+Irusta%2 C+Miriam Data will be made available on request.

## Abbreviations

AQP4: Aquaporin 4
CD11c+: a specific microglial cell type
CC: Corpus callosum
WT: wild type
KO: AQP4 knock out mouse
OPCs: oligodendrocytes precursor cells
qRT-PCR: quantitative reverse transcription polymerase chain reaction
GFAP: glial fibrillary acidic protein
OLIG2: oligodendrocyte marker
PDGFRa: Platelet-derived growth factor receptor A
OLs: mature oligodendrocytes
P11: day postnatal 11
GS: gene sets
NES: normalysed enrichment score
FDR: false discovery rate
TEM: transmission electron microscopy
Sema: semaphorins
