# Supplementary material for: The postnatal expression of transcripts and proteins in the corpus callosum, as well as its myelinization, is affected by the congenital absence of AQP4
Source: J Physiol Biochem. 2026 Mar 31;82(1):33. doi: 10.1007/s13105-026-01173-3 (PMC13038659; doi:10.1007/s13105-026-01173-3)
Supplement: Supplementary file 1 — (DOCX 60.0 KB) [file 13105_2026_1173_MOESM1_ESM.docx]

| Gene ID | Gene Symbol | Fold Change | P Value |
| --- | --- | --- | --- |
| **Semaphorins** | | | |
| **NM_172537** | **Sema6d** | **1,43** | **0,004***** |
| **NM_01135** | **Sema7a** | **-1,32** | **0,01**** |
| **NM_001281880** | **Sema4d** | **-1,51** | **0,03*** |
| NM_013659 | Sema4b | 1,28 | 0,06 |
| NM_001243072 | Sema3a | -1,2 | 0,06 |
| NM_011348 | Sema3e | -1,24 | 0.06 |
| NM_001163489 | Sema4a | -1,27 | 0,07 |
| NM_001025379 | Sema3g | 1,23 | 0,07 |
| **Plexins** | | | |
| **NM_001159521** | **Plxnb2** | **2,01** | **0,0002***** |
| **NM_018797** | **Plxnc1** | **1,86** | **0,004**** |
| **NM_008883** | **Plxna3** | **1,37** | **0,02*** |
| **Neuropilin** | | | |
| **NM_001077403** | **Nrp2** | **1,44** | **0,04*** |

**Supplementary Table S1. * p<0.05; ** p<0.01 and *** p<0.001**
