# Supplementary figures and images for: The postnatal expression of transcripts and proteins in the corpus callosum, as well as its myelinization, is affected by the congenital absence of AQP4
Source: J Physiol Biochem. 2026 Mar 31;82(1):33. doi: 10.1007/s13105-026-01173-3 (PMC13038659; doi:10.1007/s13105-026-01173-3)

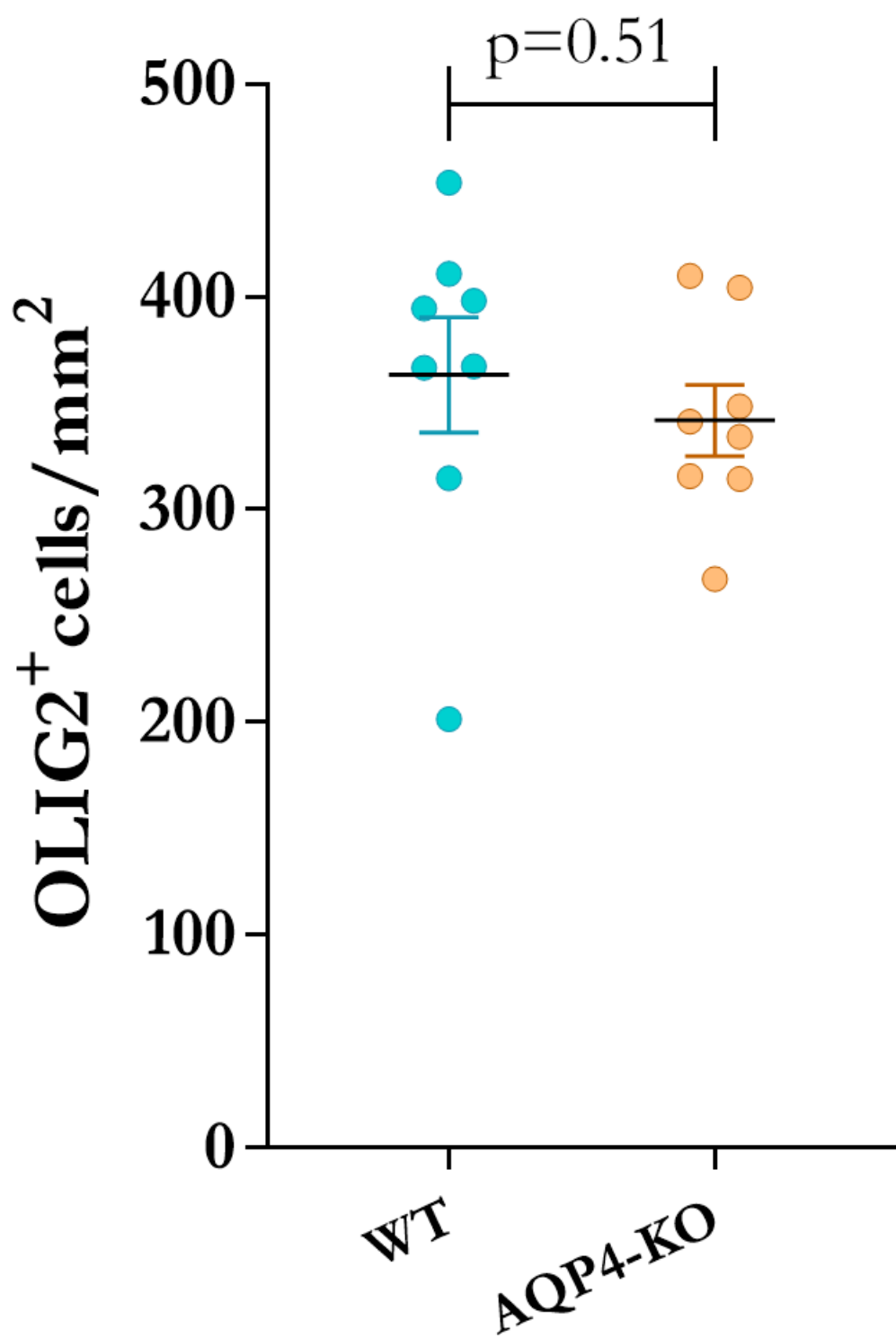

Supplement: Supplementary file 2 — (PDF 44.3 KB) Quantification of total OLIG2⁺ cells in the corpus callosum of WT and AQP4-KO mice at P11. Graph showing the mean ± SEM of the total number of OLIG2⁺ cells (including OPCs and mature oligodendrocytes) per mm² in the corpus callosum of WT and AQP4-KO mice at P11. No significant differences were found between genotypes (WT: 361 ± 18 vs KO: 355 ± 21 cells per mm²; n = 8 animals per group; unpaired Student’s t-test). [file 13105_2026_1173_MOESM2_ESM.pdf]

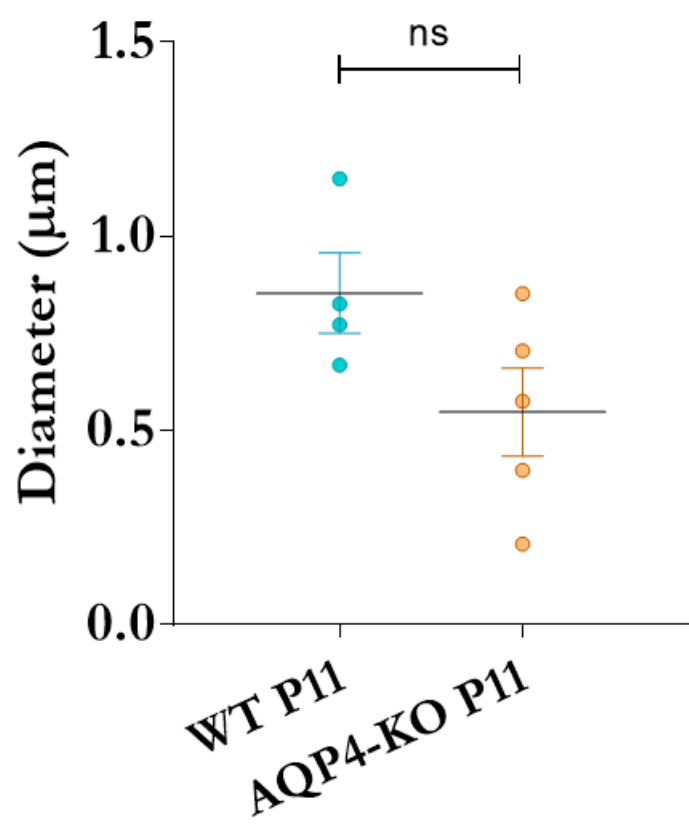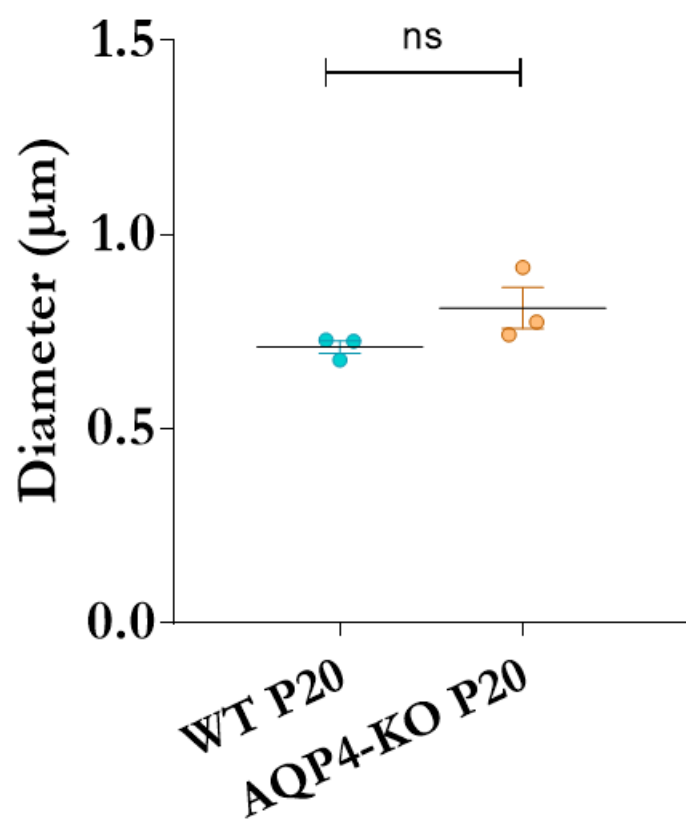

Supplement: Supplementary file 3 — (PDF 35.5 KB) Quantification of axonal diameter in the corpus callosum of WT and AQP4-KO mice. Plots showing the mean ± SEM for total axonal diameters at P11 and P20. No significant differences were detected between WT and AQP4-KO mice in either age analyzed (P11 and P20), indicating that axonal size remain unaltered in the absence of AQP4. No significant differences were found between genotypes (WT vs KO, n = 3-5 animals per group; unpaired Student’s t-test). [file 13105_2026_1173_MOESM3_ESM.pdf]
